# Dynamic Changes in Non-Volatile Components during Steamed Green Tea Manufacturing Based on Widely Targeted Metabolomic Analysis

**DOI:** 10.3390/foods12071551

**Published:** 2023-04-06

**Authors:** Anhui Gui, Shiwei Gao, Pengcheng Zheng, Zhihui Feng, Panpan Liu, Fei Ye, Shengpeng Wang, Jinjin Xue, Jun Xiang, Dejiang Ni, Junfeng Yin

**Affiliations:** 1 Key Laboratory of Tea Biology and Resources Utilization, Ministry of Agriculture, Tea Research Institute, Chinese Academy of Agricultural Sciences, Hangzhou 310008, China; 2 Key Laboratory of Horticulture Plant Biology, Ministry of Education, College of Horticulture and Forestry Sciences, Huazhong Agricultural University, Wuhan 430070, China; 3 Key Laboratory of Tea Resources Comprehensive Utilization (Co-Construction by Ministry and Province), Ministry of Agriculture and Rural Affairs, Fruit and Tea Research Institute, Hubei Academy of Agricultural Sciences, Wuhan 430064, China; 4 Enshi Tujia and Miao Autonomous Prefecture Academy of Agricultural Sciences, Enshi 445002, China

**Keywords:** Enshiyulu tea, green tea, non-volatile components, steaming, fixation, metabolomic

## Abstract

Steamed green tea has unique characteristics that differ from other green teas. However, the alteration patterns of non-volatile metabolites during steamed green tea processing are not fully understood. In this study, a widely targeted metabolomic method was employed to explore the changes in non-volatile metabolites during steamed green tea processing. A total of 735 non-volatile compounds were identified, covering 14 subclasses. Of these, 256 compounds showed significant changes in at least one processing step. Most amino acids, main catechins, caffeine, and main sugars were excluded from the analysis. The most significant alterations were observed during steaming, followed by shaping and drying. Steaming resulted in significant increases in the levels of most amino acids and their peptides, most phenolic acids, most organic acids, and most nucleotides and their derivates, as well as some flavonoids. Steaming also resulted in significant decreases in the levels of most lipids and some flavonoids. Shaping and drying caused significant increases in the levels of some flavonoids, phenolic acids, and lipids, and significant decreases in the levels of some amino acids and their peptides, some flavonoids, and some other compounds. Our study provides a comprehensive characterization of the dynamic alterations in non-volatile metabolites during steamed green tea manufacturing.

## 1. Introduction

Globally, tea is the most popular aromatic beverage after water due to its appealing flavor and potential health benefits, such as its antioxidant capacity and use for weight management, mental relaxation, and neuroprotection [1]. In China, six types of tea are produced, namely green, black, oolong, white, yellow, and dark teas, which are categorized based on their manufacturing process and sensory quality [2]. Among the six types of tea, green tea is the most widely produced and consumed tea in China, accounting for over 60% of tea production in 2021 (data from China Tea Marketing Association). Green tea is known for its umami and mild-astringent taste, fresh or chestnut aroma, green liquor color, and green appearance. The tea’s taste, health benefits, liquor color, and appearance are generally determined by its non-volatile components. The main non-volatile components in green tea are catechins, caffeine, free amino acids, flavone and flavonol glycosides, phenolic acids, soluble saccharides, and lipids, among others [3,4]. These non-volatile components are affected by many factors such as the tea variety and cultivar [5,6], harvesting season [7,8], culture measures [9,10], climatic conditions [11], maturity degree of the fresh leaves [12], and manufacturing processes [3,13].

The basic manufacturing processes of green tea include fixing, rolling, and drying [2]. Fixing is a vital step that determines the specific appearance and flavor quality of green tea [14]. Due to the inactivation effect of enzymes caused by fixation, green tea is considered a non-fermented tea, and its quality components are most similar to those of fresh leaves compared to other kinds of tea. Rolling can break the leaf cells and improve the taste concentration of green tea. Drying accelerates water loss, which is beneficial for storage and transport, and greatly increases the compounds with floral and roasted aromas [15]. Studies show that various non-volatile components change significantly during green tea processing, including catechins, free amino acids, theaflavins, flavone glycosides, sugars, alkaloids (caffeine), and lipids [4,16,17,18,19]. In addition to the non-volatile compounds, aroma compounds also result in significant changes during green tea processing [20,21].

Green teas are classified into roasted and steamed green tea according to the difference in the fixing method [22]. The fixing processes use metal/steam heat conduction. The appearance, taste, and aroma of steamed green tea differ from those of roasted green tea [23]. Although the objectives of steaming and roasting are the same—terminating the activity of enzymes—the differences between these two types of tea are believed to be due to the effects of the two fixing methods on the various quality components. Han et al. found that the roasting method resulted in higher levels of four epi-catechins in green tea compared to the steaming method [24]. Wang et al. [22] and Shi et al. [25] found that steamed green tea differed significantly from roasted green tea in the levels of catechins, free amino acids, phenolic acids, flavone glycosides, and lipids. In addition, Shi et al. found that the volatile compounds in steamed green tea differed significantly from those in roasted green tea [25,26]. The non-volatile compounds in roasted green tea, including their changes during processing, have been extensively studied [4,16,17,18,19], whereas those in steamed green tea have not been well-researched until now.

Enshiyulu tea is a typical steamed green tea that has been produced for over 1000 years in China. Due to its special flavor, which differs from that of roasted green tea, Enshiyulu tea has recently attracted the attention of tea consumers and researchers. In this study, Enshiyulu tea is used to explore the changes in non-volatile metabolites during steamed green tea processing using a widely targeted metabolomic method. The results of our study provide novel insights into the formation mechanism of the qualities of steamed green tea.

## 2. Materials and Methods

### 2.1. Chemicals and Reagents

Deionized water was obtained using a Milli-Q water purification system (Millipore, Billerica, MA). Mass-spectral-grade methanol, acetonitrile, and ethanol were obtained from Merck Corporation (Darmstadt, Germany). Standard compounds were obtained from Sigma Corporation (St. Louis, MO, USA) and BioBioPha Co., Ltd. (Kuming, China).

### 2.2. Tea Samples

**Figure 1:**
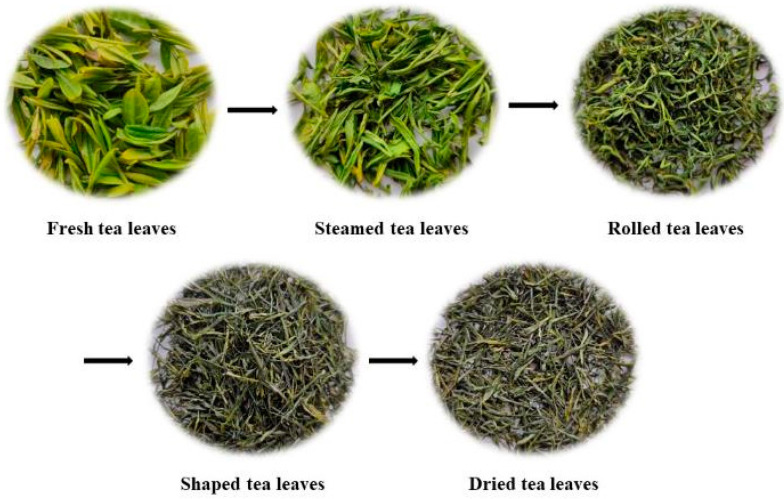
The appearance of tea leaves after the various manufacturing processes.

About 100 kg of fresh tea leaves of Clonal Longjing 43 cultivar (Camellia sinensis [L] O. Kuntze) was obtained from the tea gardens of Jinfeng tea Co., Ltd. (Enshi, China) on 30 March 2022 for use in our study. Fresh tea leaves were one bud with two leaves. After naturally withering for about 4 h at 20–25 °C on bamboo sieves, the fresh tea leaves were continuously fixed with steam and hot air for 40–50 s using a steam-hot-air fixation machine (6CZQRC-300, Sichuan Dengyao Machinery Equipment Co., Ltd., Chengdu, China). The steam pressure was 3.8–4.0 Mpa and the temperature of the hot air was 520–550 °C. The fixed tea leaves were subsequently dehydrated for 40–50 s at 150–180 °C prior to rolling. The rolling was performed using a 6CR-55 rolling machine (Hubei Tianchi Machines Co., Ltd., Yichang, China) and the rolling time was 40 min. The rolled tea leaves were then subjected to shaping. The shaping process consisted of three steps: (a) the rolled tea leaves were first dried to about 45% moisture at 100–120 °C; (b) the preliminarily dried tea leaves were shaped using a shaping machine (6CL-12/80, Sichuan Dengyao Machinery Equipment Co., Ltd., Chengdu, China) at 90–110 °C for about 30 min; and (c) the tea leaves were further shaped using a fine shaping machine (60K-S, Zhejiang Kawasaki Tea Machinery Co., Ltd., Hangzhou, China) at 90 °C for about 45 min to 25% moisture. Finally, the shaped tea leaves were dried at 90–100 °C for 20 min until the moisture was below 6%. The tea’s appearance after the various manufacturing processes is shown in Figure 1.

After each process, samples of fresh (FTL), steamed (STL), rolled (RTL), shaped (SPTL), and dried (DTL) tea leaves were collected after each step. The samples were immediately frozen in liquid nitrogen and then freeze-dried (FD 5–10; SIM International, Co., USA) at –50 °C for 48 h. Three replicate samples were collected simultaneously from different points during a single process. In addition, equal amounts of each tea sample were mixed to prepare quality control (QC) samples. The tea samples were stored at –20 °C until further analysis. 

### 2.3. Widely Targeted Metabolomic Analysis

Widely targeted metabolomic analysis was performed with the assistance of the MetWare (http://www.metware.cn/, accessed on23 September 2022 ) corporation (Wuhan, China), a professional institution for metabolomic analysis. The tea samples were ground into a homogeneous powder using a mortar and pestle prior to extracting the non-volatile compounds. A precise amount of tea powder (100 mg) was extracted with 10 mL of 70% methanol. The solutions were then centrifugated at 10,000× *g* for 10 min. The resulting suspensions were filtered (SCAA-104, 0.22-μm pore size; ANPEL, Shanghai, China, http://www.anpel.com.cn/ (accessed on10 September 2022)) prior to UPLC–MS/MS analysis.

The extracted non-volatile compounds were separated using an ultra-performance liquid chromatography system (UPLC; Shim-pack UFLC Shimadzu CBM30A, Kyoto, Japan), which had an Agilent SB-C18 column (1.8 μm, 2.1 mm × 100 mm, Foster City, CA, USA). The elution solvents A and B consisted of deionized water containing 0.1% formic acid and pure acetonitrile, respectively. The elution profiles were as follows: 0 min, 5% B; 0–9 min, linearly increased to 95% B, 9–10 min, 95% B; 10–11.10 min, linearly decreased to 5% B; 11.10–14 min, 5% B. The chromatography column was invariably maintained at 40 °C and the injection volume was 4 μL.

The detection of non-volatile compounds was performed using a triple-quadrupole-linear ion trap/orbitrap mass spectrometer (QQQ-LTQ-Orbitrap-MS, API 4500 QTRAP system, Applied Biosystems; Carlsbad, CA, USA), which had an ESI turbo ion-spray interface. Prior to detection, amounts of 10 and 100 μmol/L of polypropylene glycol were used to conduct instrument tuning and mass calibration in QQQ and LIT modes, respectively. Positive and negative MS ion modes were used in both. The ion-spray (IS) voltage for positive mode was 5500 V and the IS voltage for negative ion mode was −4500 V. The source temperature was set at 550 °C. The ion source gas I (GSI) and GSII were 50 psi and 60 psi, respectively. The QQQ scan parameters were acquired beforehand in MRM mode using a series of standards. The declustering potential (DP) and collision energy (CE) were determined and optimized for individual MRM transitions. The mass scan range was 100–1000 m/z and the resolution of the orbit trap was 30,000.

The raw data files of all samples were processed using Sieve software (Thermo Fisher Scientific; Waltham, MA, USA). For the peak picking and alignment, the mass width was 0.02 Da and the retention-time (RT) width was 0.2 min. The retention time (RT) and MS information of each chromatographic peak were compared to the MetWare MS database to identify the compounds.

### 2.4. Data Analysis

Principal component analysis (PCA) was performed using Simca-P 14.0 software (Umetrics AB, Umeå, Sweden) and the data used for PCA were Pareto-scaled (the data were centralized and then divided by the square root of the standard deviation). The filter criteria for the differential metabolites between the two groups were as follows: the *p*-value was less than 0.01 (*t*-test) and the fold change was greater than 1.2 or less than 0.83. Data were UV-scaled and then heat maps were created using MultiExperiment Viewer 4.8.1 software (Oracle Corporation, Redwood, CA, USA). The Venn and K-Means graphs were generated using R online software (http://www.r-project.org/ (accessed on 23 September 2022)). 

To perform the enrichment analysis using the Kyoto Encyclopedia of Genes and Genomes (KEGG), the significantly changed metabolites were first annotated using the KEGG Compound database (http://www.kegg.jp/kegg/compound/ (accessed on 23 September 2022)). Then, the annotated metabolites were mapped to the KEGG Pathway database (http://www.kegg.jp/kegg/pathway.html (accessed on 23 September 2022)). The corresponding mapped pathways were then fed into a metabolite sets enrichment analysis (MSEA). The significance values of the KEGG pathways were calculated using a hypergeometric test.

## 3. Results and Discussion

### 3.1. Overall Characterization of Non-Volatile Compounds

The non-volatile compounds were determined using a widely targeted metabolomic method. After comparing the RT and MS information of each peak to those in the MetWare MS database, a total of 735 non-volatile components were identified, including 77 amino acids and their derivative compounds, 180 flavonoids, 104 phenolic acids, 19 tannins, 42 nucleotides and their derivative compounds, 48 alkaloids, 54 organic acids, 54 saccharides, 116 lipids, 10 lignans, 5 coumarins, 8 terpenoids, 13 vitamins, and 5 other compounds (Table S1). 

**Figure 2:**
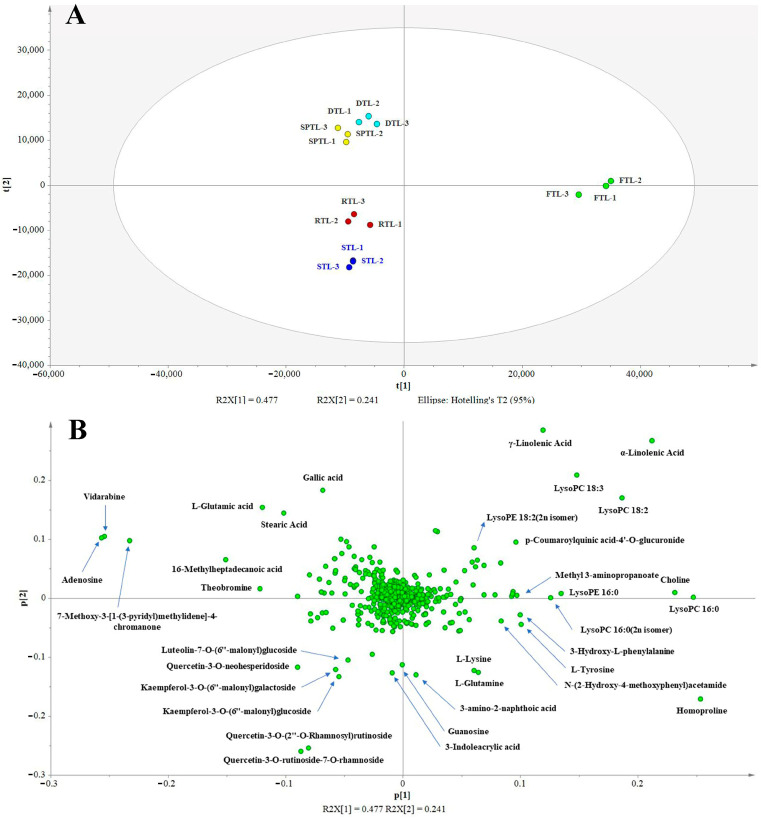
Principal component analysis score (**A**) and loading (**B**) plot of tea samples. R^2^X = 0.718, Q^2^ = 0.601. The data were Pareto-scaled.

To obtain an overview of the changes in the non-volatile compounds during the manufacturing processes of steamed green tea, PCA was performed using the peak areas of the 735 identified compounds. Firstly, the reproducibility of the metabolomics analysis was assessed using QC samples [27]. Three QC samples were clustered closely in the center of the PCA score plot (Figure S1), which indicates that the metabolomics analysis used in our study had good reproducibility. As shown in Figure 2A, the first two principal components explained 71.8% of the total variance (R^2^X) and had a high predictive ability (Q^2^ = 60.1%), indicating that this model showed good fit and predictability. The tea samples from the same processing step were well-clustered, whereas the tea samples from different processing steps were clearly separated, proving that there were significant differences in the non-volatile compounds between the FTL and DTL tea samples. The most significant alterations of the non-volatile compound were triggered by the steaming process in the PC1 direction, and further alterations mainly occurred after the shaping process in the PC2 direction (Figure 2A). Thus, these five tea samples could be classified into three groups, namely the FTL group, STL and RTL group, and SPTL and DTL group. In addition to steaming green tea, Wang et al. also observed similar results in the processing of roasted green tea [19]. Interestingly, the appearance of the tea leaves changed significantly after steaming and shaping (Figure 1). Thus, the changes in the non-volatile compounds were consistent with the alterations in the appearance of the tea leaves during the processing of steamed green tea. To further identify important compounds that could distinguish these tea samples, a PCA loading plot was generated (Figure 2B). The non-volatile compounds that noticeably contributed to the differences between the tea samples were adenosine, vidarabine, 7-methoxy-3-[1-(3-pyridyl)methylidene]-4-chromanone, homoproline, α-linolenic acid, γ-linolenic acid, LysoPC 16:0, choline, LysoPC 18:2, quercetin-3-O-rutinoside-7-O-rhamnoside, L-glutamic acid, quercetin-3-O-(2″-O-rhamnosyl)rutinoside, quercetin-3-O-neohesperidoside, and gallic acid.

### 3.2. Screening of Significantly Altered Non-Volatile Compounds in Each Processing Step

**Figure 3:**
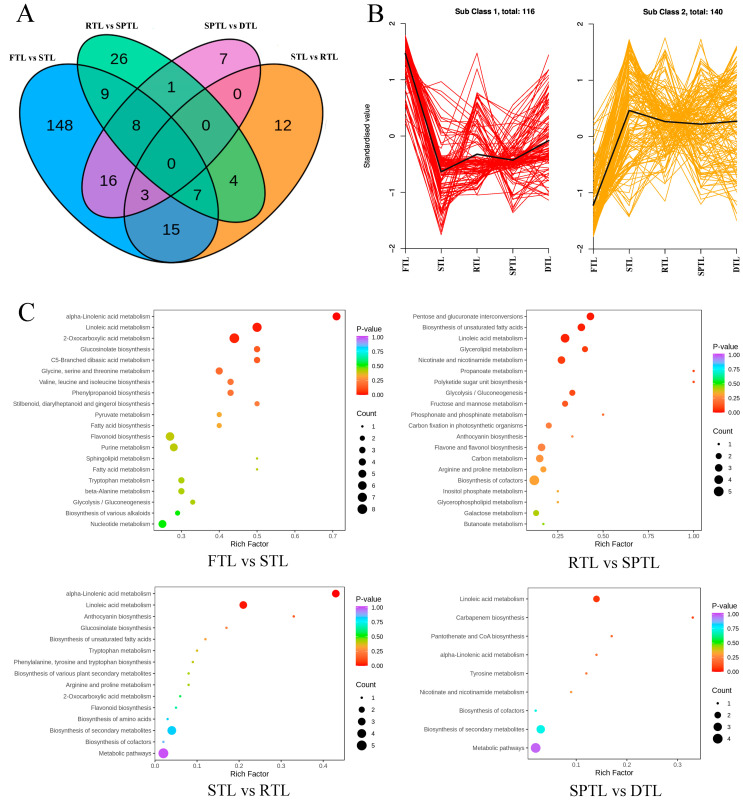
Difference analysis of non-volatile compounds. (**A**) Venn plot. (**B**) K-means clustering plot. (**C**) KEGG enrichment pathways. The *Y*-axis represents the KEGG pathway and the *X*-axis represents the “enrich enrichment factor”, which is the ratio of the DEP number to the total number of annotated proteins in each pathway.

To better understand the influence of each processing step on the changes in the non-volatile compounds, the differential metabolites were screened using two criteria: *p* < 0.01 (*t*-test) and fold change values greater than 1.20 or less than 0.83. In total, 256 non-volatile compounds showed significant changes in at least one processing step (Figure 3A and Table S2). In terms of the different processing steps, steaming resulted in the highest number of differential compounds, with up to 206 compounds significantly changed (109 up, 97 down). Rolling, shaping, and drying resulted in 41 (30 up, 11 down), 55 (33 up, 22 down), and 35 (30 up, 5 down) significantly changed compounds, respectively (Figure 3A and Table S2). Wang et al. also found that the changes in the non-volatile compounds mainly occurred in the fixing step compared to the rolling and drying steps in the processing of roasted green tea [19]. This proves that the fixing step is vital to the formation of high-quality green tea. The differential compounds were divided into two subclasses using K-means clustering (Figure 3B). Subclass 1 contained 116 compounds, which decreased significantly after steaming. Subclass 2 contained 140 compounds, which displayed converse trends. To understand the internal relationships between the significantly changed non-volatile compounds, we performed a KEGG pathway enrichment analysis. As shown in Figure 3C, some pathways were closely related to the biosynthesis or metabolism of important flavor components such as alpha-linolenic acid metabolism (ko00592); linoleic acid metabolism (ko00591); pentose and glucuronate interconversions (ko00040); glycine, serine and threonine metabolism (ko00260); biosynthesis of unsaturated fatty acids (ko01040); valine, leucine and isoleucine biosynthesis (ko00290); phenylpropanoid biosynthesis (ko00940); glycerolipid metabolism (ko00561); glycolysis/gluconeogenesis (ko00010); fructose and mannose metabolism (ko00051); and anthocyanin biosynthesis (ko00942). Among these, pentose and glucuronate interconversions (ko00040); glycine, serine, and threonine metabolism (ko00260); phenylpropanoid biosynthesis (ko00940); and glycolysis/gluconeogenesis (ko00010) are involved in the accumulation of important taste compounds, including amino acids, flavonoids, saccharides, and organic acids, among others [27,28]. Alpha-linolenic acid metabolism (ko00592) and linoleic acid metabolism (ko00591) are related to the production of volatile aliphatic compounds, which are responsible for the green, fresh, and/or grassy scent [29].

**Figure 4:**
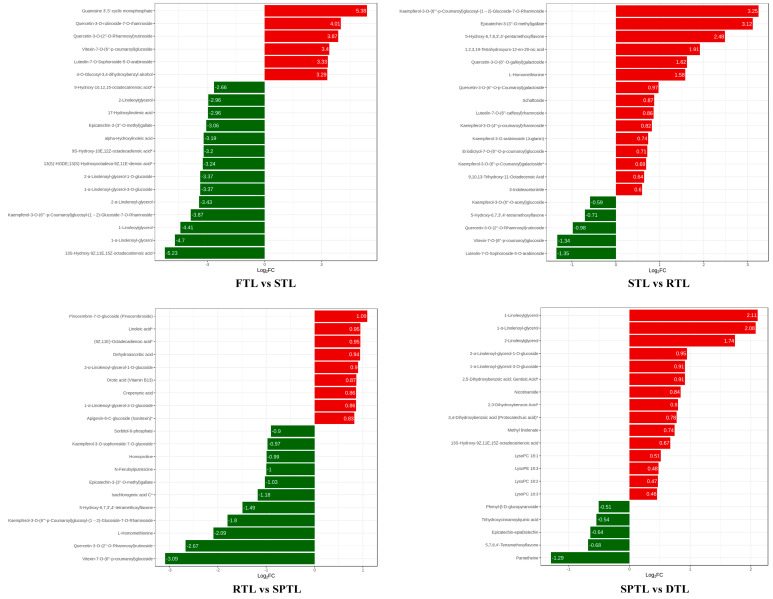
Bar chart of top fold changes in the non-volatile compounds. FC: fold change. “*” indicates the presence of isomers of the substance.

To further investigate each processing step in the accumulation of non-volatile compounds, we screened and compared these top differential compounds (TDCs) with the high fold changes and the results are shown in Figure 4. The TDCs were highly diverse among the different comparison groups. In the FTL vs. STL group, the upregulated TDCs were mainly flavonoids, along with nucleotides and their derivatives, whereas the downregulated TDCs were mainly lipids. It has been reported that most lipids exhibit a sharp decline after fixing with a roasting method [4]. In the STL vs. RTL group, most TDCs significantly increased, mainly flavone and flavonol glycosides. The results of a previous study, in which the contents of naringenin, kaempferol, and apigenin glycosides reached their maximum during rolling, are consistent with our findings [30]. In the RTL vs. SPTL group, the increased TDCs included lipids, flavonoids, and other compounds, and the decreased TDCs mainly included flavonoids. Similar to the STL vs. RTL group, most TDCs in the SPTL vs. DTL group were significantly upregulated. These were mainly lipids, including 1-linoleoylglycerol, 1-α-linolenoyl-glycerol, and 2-linoleoylglycerol, and D-panose, and epicatechin-3-(3″-O-methyl)gallate, among others. Overall, the fold changes of the TDCs in the FTL vs. STL group were significantly higher than those in the other groups. This observation was expected because fixing is the process that significantly influences the flavor of green tea. 

### 3.3. Dynamic Changes in Differential Non-Volatile Compounds

#### 3.3.1. Amino Acids and their Derivatives

**Figure 5:**
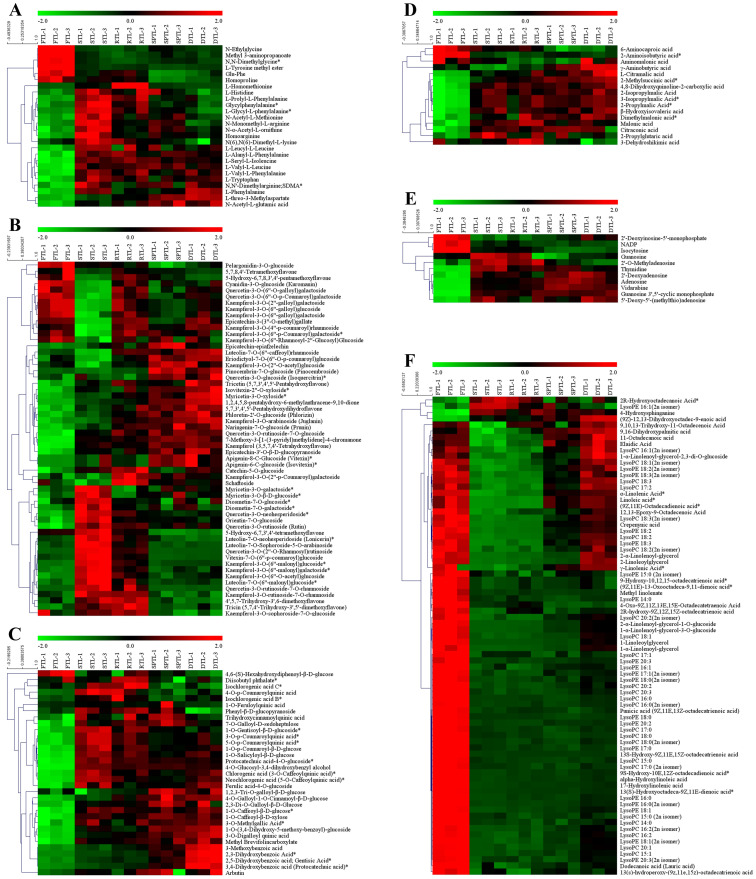
Heat map of the levels of differential non-volatile compounds throughout the processing period. The data were UV-scaled and clustered according to Pearson correlation coefficients. (**A**) Amino acids and their derivatives; (**B**) flavonoids; (**C**) phenolic acids; (**D**) organic acids; (**E**) nucleotides and their derivatives; (**F**) lipids. “*” indicates the presence of isomers of the substance.

Amino acids significantly influence the taste and aroma of tea. In total, 26 amino acids, including 20 proteinaceous and 6 non-proteinaceous amino acids, have been identified in tea to date [31]. They have an umami, bitter, or sweet taste in water [31]. High amounts of free amino acids are essential for the freshness and mellowness of green tea [32]. In addition to individual patterns, amino acids can be condensed to form oligopeptides via an amide bond. Oligopeptides are water soluble and have similar taste properties to free amino acids. Oligopeptides have been reported to exhibit various tastes such as umami, sweet, bitter, and astringent [33]. Similar to roasting [19], we found that steaming resulted in increasing levels of most differential amino acids, oligopeptides, and other derivatives (Figure 5A). The increase in amino acids and oligopeptides is believed to be on account of the protein degradation induced by heat and humidity [34]. During the next processing steps, these decreased amino acids and their derivatives remained stable. For the increased amino acids and derivatives, L-histidine, L-prolyl-L-phenylalanine, and homoarginine, among others, showed decreasing trends, whereas L-alanyl-L-phenylalanine, L-tryptophan, and L-phenylalanine showed stable or increasing trends (Figure 5A). 

#### 3.3.2. Flavonoids

Flavonoids are secondary metabolites that are found in many plants and consist of catechins (flavanols), flavones, flavonols, anthocyanidins, and leucoanthocyanidins. We found that mainly the flavone and flavonol glycosides changed significantly (Figure 5B). We observed three trends for the differential flavonoids: (1) the levels of cyanidin-3-O-glucoside, quercetin-3-O-(6″-O-galloyl)-galactoside, and epicatechin-3-(3″-O-methyl)gallate exhibited wave-line trends; (2) the levels of luteolin-7-O-(6″-caffeoyl)rhamnoside, quercetin-3-O-glucoside, and quercetin-3-O-rutinoside-7-O-glucoside showed significant increases during the shaping and drying steps; and (3) the levels of myricetin-3-O-β-D-glucoside, quercetin-3-O-rutinoside-7-O-rhamnoside, and kaempferol-3-O-(6″-malonyl)glucoside showed significant increases after steaming and then significant decreases during the next steps. It has been reported that the levels of most flavones, flavonols, and their glycosides significantly increase during the manufacturing of green tea [19], whereas another study demonstrated that the levels of flavone and flavonol glycosides slightly decreased [18]. The contradictions between our study and previous studies may be due to the chemical changes, such as isomerism, hydrolysis, and the partial oxidation polymerization of flavone and flavonol glycosides, that simultaneously exist in the heat treatment process, leading to variations in composition [30,35]. Flavone and flavonol glycosides contribute to tea’s astringent taste and infusion color [19]. The alterations in the contents of flavone and flavonol glycosides can affect the quality of green tea.

Catechins are the most important flavonoids in fresh tea leaves and contribute significantly to the astringency and bitter taste of green tea [36]. Due to the inactivation effect of steaming on oxidation and hydrolysis enzymes, we found that the catechins remained relatively stable during the processing of steamed green tea (Figure 5B, Table S1), which is consistent with the processing of roasted green tea [19]. The relative stationarity of catechins during green tea processing indicates that the levels of catechins in green tea are dominated by fresh tea leaves. 

#### 3.3.3. Phenolic Acids

Phenolic acids are a group of aromatic compounds that contain a phenolic skeleton and carboxyl acid. Phenolic acids also contribute significantly to the taste of tea. The composition and level of phenolic acids in foods are affected by many factors during processing, including thermal treatment, pressing, enzyme treatment, fermentation, etc. [37]. In our study, most phenolic acids showed significant increases after thermal treatments, such as steaming, shaping, and drying (Figure 5C). The levels of chlorogenic acid, isochlorogenic acid B, isochlorogenic acid C, and 4-O-p-coumaroylquinic acid significantly increased after steaming, and the levels of 1,2,3-tri-O-galloyl-β-D-glucose, 1-O-caffeoyl-β-D-xylose, 3-methoxybenzoic acid, and 2,3-dihydroxybenzoic acid significantly increased during shaping and drying. In addition, gallic acid significantly increased (*p* < 0.05, Table S1) in our study The accumulation of gallic acid may originate from the degradation of gallated catechins [38,39]. Phenolic acids, including gallic acid, chlorogenic acid, caffeic acid, and 5-O-caffeoylquinic acid, are responsible for the bittern and astringent taste [37,40]. Thus, an increase in phenolic acid levels may have a negative effect on the taste of green tea. 

#### 3.3.4. Organic Acids

Organic acids are an essential group of compounds in teas that contribute to the sourness and mild astringence, as well as the health-promoting properties, of tea, as shown in numerous studies [41,42]. As shown in Figure 5D, we found that most organic acids, including L-citramalic acid, malonic acid, and 2-methylsuccinic acid, displayed significant increases, except for 6-aminocaproic acid, 2-aminoisobutyric acid, and aminomalonic acid. In addition, citric acid, L-malic acid, and maleic acid, the three main organic acids in tea, showed significant increases (*p* < 0.05, Table S1) in our study. High acidity is generally considered a disadvantage in the taste of tea, especially green tea [41]. The sourness associated with the increase in organic acid levels may negatively affect the taste of steamed green tea. However, the levels of citric acid, anechoic acid, and quinic acid significantly increased during the production process of roasted green tea (*p* < 0.05), whereas that of L-malic acid exhibited a significant decrease after fixation, which may help balance the taste of roasted green tea [43].

#### 3.3.5. Nucleotides and Their Derivatives

Nucleotides and their derivatives (mainly nucleosides and nucleobases) are the precursors of nucleic acids and have been demonstrated to affect the immune, nervous, metabolic, and hepatic systems [44]. It has been shown that fixing is the key to enhancing the levels of most nucleotides and their derivatives [19], which we also observed in our study (Figure 5E and Table S1). However, the nucleotides and derivatives exhibited an upward trend during the processing of roasted green tea [42]. Overall, the levels of 2′-deoxyinosine-5′-monophosphate, NADP, and isocytosine decreased, and the levels of adenosine, vidarabine, and guanosine 3′,5′-cyclic monophosphate, among others, increased during the manufacturing of steamed green tea. In addition, the levels of adenosine 5′-monophosphate (AMP) and uridine 5′-monophosphate significantly increased (*p* < 0.05, Table S1). The degradation of RNA is believed to account for increases in the levels of nucleosides and nucleotides [27]. Adenosine has been shown to selectively enhance the sweet taste through A2B receptors in the taste buds [45]. In addition, AMP are known as an umami enhancer [44]. The higher accumulation of adenosine and AMP may play a significant role in the formation of green tea taste.

#### 3.3.6. Lipids

Lipids are essential components that play a role in a series of physiological activities in plants. With regard to tea flavor, unsaturated fatty acids, such as linoleic acid, α-linolenic acid, and palmitoleic acid, act as aroma precursors for volatile aliphatic alcohols, aldehydes, and lactones. These compounds contribute to the green and/or fresh odors of tea via oxidation and degradation [46]. In our study, the lipids detected included free fatty acids, glycerolipids, lysophosphatidylcholines (LysoPCs), lysophosphatidylethanolamines (LysoPEs), and sphingolipids (Table S1). As shown in Figure 5F, steaming caused pronounced alterations of tea lipids and resulted in significant decreases in most lipid levels, which is in agreement with the findings of a previous study [4]. This observation is in agreement with the expectation that high and/or prolonged heat treatments typically cause the degradation of macromolecular compounds. Unexpectedly, the drying step resulted in significant increases in some lipid levels (Figure 5F). Lipids typically work with proteins to form cell membranes, and cell membranes tend to disintegrate under heat and water, which releases free lipids.

#### 3.3.7. Other Compounds

Alkaloids are a class of nitrogen-containing alkaline organic compounds commonly found in plants and animals. Among these alkaloids, purine alkaloids, such as caffeine, theobromine, and theacrine, are the most important components that contribute to the flavor and function of tea [47]. Only theacrine showed an increasing trend in our study, whereas caffeine and theobromine remained stable (Table S1). Saccharides, especially soluble sugars such as sucrose, glucose, fructose, arabinose, maltose, and galactose, are important components that contribute to the sweetness of tea. These sugars can also react with amino acids to produce aroma compounds under high temperatures [46]. We observed that the levels of maltose significantly decreased in the four processing steps; however, we observed an opposite trend in the levels of galactose t (Table S1). In addition, some lignans, coumarins, terpenoids, vitamins, and other compounds showed significant changes during the manufacturing of steamed green tea (Table S1).

## 4. Conclusions

In this study, a widely targeted metabolomic method was used to investigate the changes in non-volatile metabolites during the processing of steamed green tea. In total, 735 non-volatile compounds were identified, and 256 compounds showed significant changes during different processing steps. Steaming, followed by shaping, showed the greatest impact on the alterations of non-volatile compounds in terms of the number of fold changes of the differential compounds. However, these differential compounds excluded most amino acids, catechins, sugars, and caffeine. This suggests that the quality of steamed green tea is largely determined by the quality of the fresh tea leaves. The results of this study enhance our understanding of the chemistry of green tea manufacturing and provide new molecular targets for the quality regulation of steamed green tea. By optimizing the processing parameters to compensate for the influence of high humidity on the changes in the compounds during the steaming process, decreases in the levels of some flavonoids can be prevented.

## Data Availability

Data is contained within the article or Supplementary Material.

## Supplementary Materials

The following supporting information can be downloaded at https://www.mdpi.com/article/10.3390/foods12071551/s1. Figure S1: The score plot of principal component analysis of tea and QC samples; Table S1: List of 735 identified non-volatile compounds and their contents during the processing of steamed green tea; Table S2: List of 256 significantly changed compounds and their *p* values, fold changes (FC), and log_2_FCs during each processing step.
