# Supplementary figures and images for: Dynamic Changes in Non-Volatile Components during Steamed Green Tea Manufacturing Based on Widely Targeted Metabolomic Analysis
Source: Foods. 2023 Apr 6;12(7):1551. doi: 10.3390/foods12071551 (PMC10094149; doi:10.3390/foods12071551)

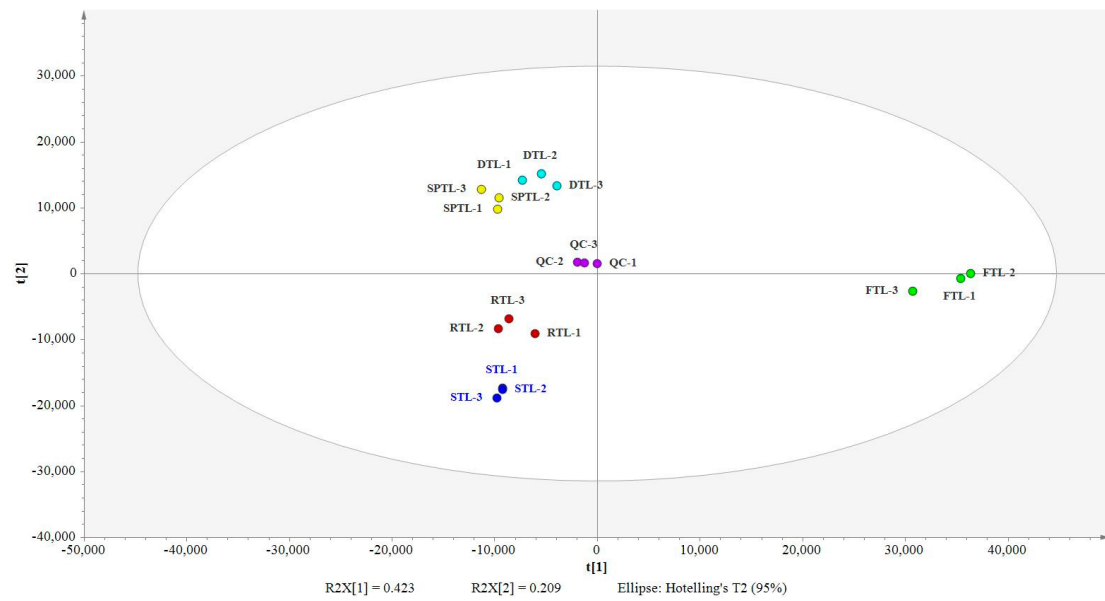

Figure S1: The score plot of principal component analysis of tea and QC samples

Supplement: Supplementary file 1 [file foods-12-01551-s001.zip › Supplementary Materials-Figure S1.pdf]
